# The roles of centrosome amplification in diabetes-associated diseases

**DOI:** 10.3389/fcell.2026.1877870

**Published:** 2026-09-21

**Authors:** Shao Chin Lee, Peng Xu, Qi Gui Wu

**Affiliations:** 1 Laboratory of Cell Biology, School of Life Sciences, Shanxi University, Taiyuan, Shanxi, China; 2 Laboratory of Cell Biology, School of Life Sciences, Jiangsu Normal University, Xuzhou, Jiangsu, China

**Keywords:** centrosome amplification, aberrant ciliogenesis, genomic instability, inflammation, diabetes-associated diseases

## Abstract

The structure and function of the centrosome and the adverse consequences of centrosome aberration are increasingly understood. Centrosome amplification (CA), probably the most studied form of centrosome abnormalities, occurs when a cell harbors more than two centrosomes. Diabetes is associated with the development of numerous diseases, the pathogenesis mechanisms of many of which, if not all, are not well understood. We have reported that diabetes triggers CA, which has pathological roles. In this context, we review the evidence that CA in diabetes promotes the development of diseases (e.g., cancer or vascular complications) via multiple mechanisms (e.g., genomic instability, inflammation, and aberrant ciliogenesis). CA is likely a mechanism underlying many, if not all, disease complications in diabetes.

## 1 Introduction

The centrosome is a non-membrane organelle in animal cells that acts as the primary microtubule- and actin-organizing center (Farina et al., 2016), with its classical function in proper chromosome segregation during cell division. Centrosomal aberrations occur in numerical and structural forms, with centrosome amplification (CA), a state in which more than two centrosomes are present in a cell, probably the most investigated type of abnormality. CA alters cell structure and function, thereby causing/favoring the development of diseases.

Diabetes affects approximately 11% of the population worldwide (Sun et al., 2022) and is associated with more than 100 complications. We used the term “diabetes-associated diseases (DAD)” rather than diabetic complications, as the number of DAD is increasing, and at least some of them (e.g., cancer and fracture) have not yet been nominated as complications by institutional authorities. Our research team has reported that CA is increased in cells treated with pathophysiological factors in diabetes, diabetic animal models, and human patients, via various signaling pathways (Zhao et al., 2021; Zhao et al., 2026). Against this background, we provide evidence that CA in diabetes (CAID) plays roles in the pathogenesis of DAD, using cancer, vascular complications, and mechanisms of genomic instability, chronic inflammation, and aberrant ciliogenesis as examples (Figure 1).

**FIGURE 1:**
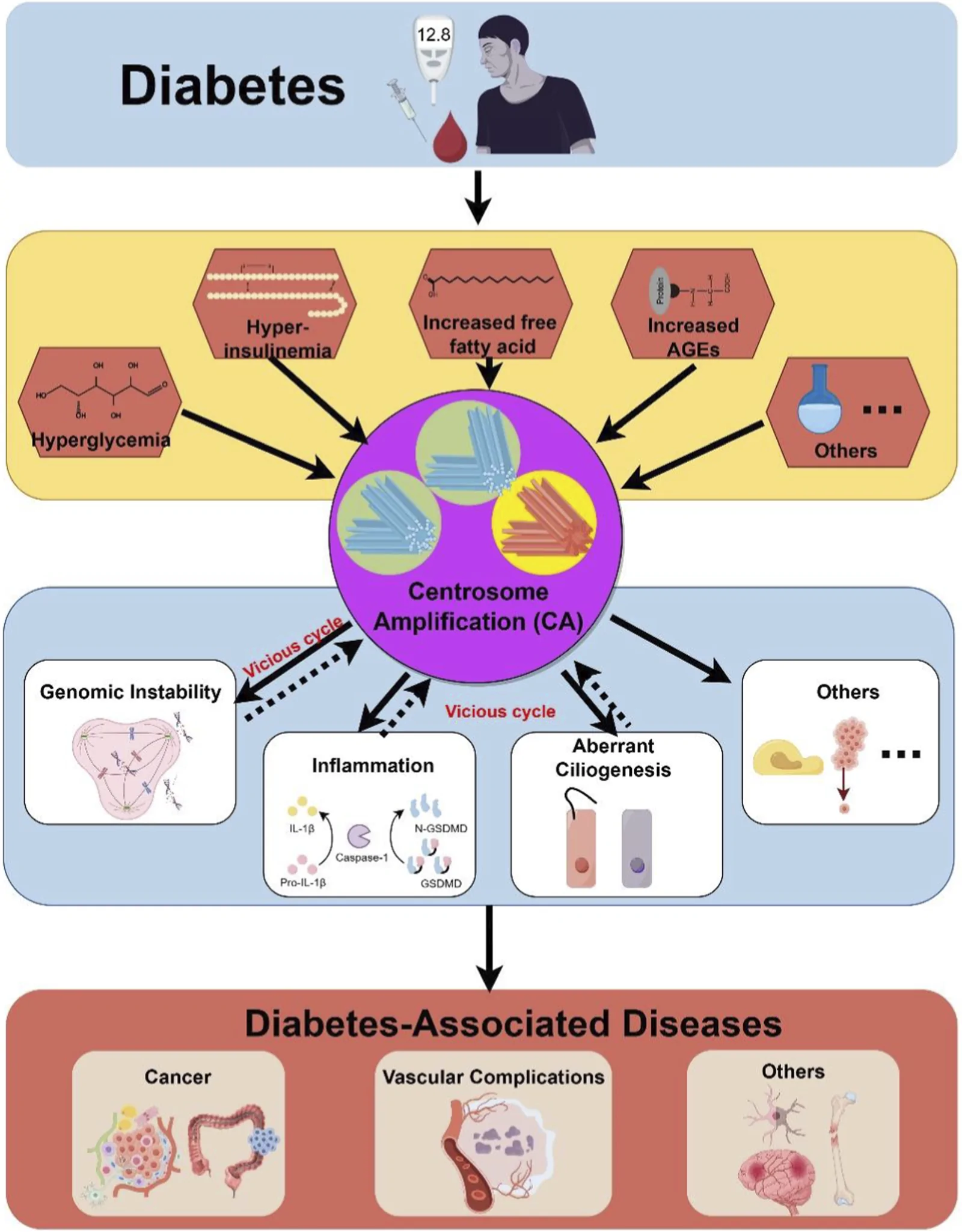
The roles of CA in diabetes-associated cancer and vascular disease. The upper panel depicts diabetes as the pathological condition that initiates the development of complicated diseases. The middle panel shows that the pathophysiological factors in diabetes trigger centrosome amplification. The circle in the center represents centrosome amplification. The lower middle panel illustrates three representative downstream consequences of centrosome amplification. The bottom panel lists two common diseases caused by centrosome amplification. Dashed arrows denote feedback actions.

The centrosome regulates many biological events, including cell morphology, replication, polarity, transition, secretion, formation of structures (e.g., the immune synapse), and the transportation and localization of molecules and organelles, all of which can be disturbed by centrosome aberrations and thereby favor pathogenesis. More specifically, CA can cause errors in chromosome segregation, DNA fragmentation, and micronucleus formation due to replication stress, as well as point mutations, which represent different forms of genomic instability. In addition to proinflammatory cytokines, CA can promote the secretion of other bioactive molecules such as lipoprotein (a) (Lee et al., manuscript in preparation), suggesting that CA-triggered cell secretion may be implicated in diverse pathological processes. Ciliogenesis initiates from the mother centrosome, which provides the foundation for integrating ciliary structural components. CA inhibits ciliogenesis via different mechanisms. Typically, it blocks/inhibits the recruitment/transport of molecules required for the formation or growth of the ciliary base.

In some cases, when other molecular/cellular events occur in the presence of CA, multiple cilia or cilia over-elongation can be seen. Abnormal ciliogenesis disrupts the sensing of environmental signals and internal signal transduction, both of which are implicated in disease. Accumulated evidence indicates that genomic instability, inflammation, and aberrant ciliogenesis play roles in cancer development and vascular diseases.

## 2 CA causes genomic instability, inflammation, and aberrant ciliogenesis

### 2.1 Genomic instability

CA induces genomic instability primarily by driving chromosomal instability, numerical aneuploidy, and structural chromosome aberrations via errors in mitotic spindle assembly and chromosome segregation (Ganem et al., 2009; Pihan, 2013). CA produces extra spindle poles, leading to multipolar mitosis and chromosome segregation errors (i.e., lagging chromosomes and unequal distribution). The daughter cells have aneuploid karyotypes (gain/loss of chromosomes). It appears that most daughter cells die when CA is high (e.g., many centrosomes per cell). Nevertheless, some cells can survive when CA occurs to a moderate extent (e.g., 3–4 centrosomes per cell). In pseudo-bipolar spindles, where extra centrosomes are clustered into two poles, a mechanism evolved to counteract CA; the cells also pass through a transient multipolar spindle intermediate, where merotelic kinetochore-microtubule attachments (one kinetochore attached to microtubules from multiple poles) can accumulate. Syntelic attachments (both sister kinetochores attached to one pole) can also increase. Thus, CA results in chromosome instability even in the absence of persistent multipolarity. Lagging chromosomes by CA often form micronuclei in daughter cells, which undergo premature replication or envelope rupture, leading to extensive chromosome shattering (Zhang et al., 2015).

Currently, there is no convincing evidence that CA directly causes mutation at the DNA sequence level. However, indirectly, lagging chromosome-produced micronuclei with defective nuclear envelopes trigger replication stress, premature chromosome condensation, and DNA double-strand breaks. Re-ligation via error-prone non-homologous end joining can introduce small indels or microhomology-mediated changes, which can co-occur with kataegis, the localized hypermutation of clustered base substitutions mediated by APOBEC3 cytidine deaminases near breakpoints (Mazzagatti et al., 2024; Ogden et al., 2017).

### 2.2 Inflammation

CA activates the PIDDosome complex, which contains PIDD1–RIPK1–NEMO/IKKγ, triggering NF-κB signaling and the secretion of chemokines and cytokines. The resulting paracrine secretome polarizes bone-marrow-derived macrophages toward an activated M1-like proinflammatory phenotype (Garcia-Carpio et al., 2023). It can also rapidly induce ROS generated by NADPH oxidase, leading to nuclear NRF2 accumulation and transcriptional upregulation of various genes. This oxidative stress drives the secretion of proinflammatory factors, described as the extra centrosomes-associated secretory phenotype (ECASP) (Arnandis et al., 2018). Indirectly, CA-caused mitotic errors (e.g., micronuclei) produce cytosolic dsDNA, which activates cGAS–STING pathways, leading to NF-κB signaling and cytokine secretion, which further fuels chronic inflammation (Pancione et al., 2021). The causative role of CA is supported by the observation that inhibiting CA (e.g., PLK4 knockdown) attenuated the secretion of proinflammatory cytokines IL-6, TNF-α, and IL-1β (Zhang et al., 2023).

### 2.3 Aberrant ciliogenesis

Aberrant primary ciliogenesis occurs in numerical (i.e., loss or increase) or structural (i.e., shortening or elongation) manners. Here, the discussion covers the numerical changes. CA prevents proper maturation of the centriole into a functional basal body. Centrioles remain sequestered in supernumerary centrosomes instead of migrating to and docking at the membrane, resulting in aciliation or drastically reduced ciliation frequency. In some cases of aciliation, cilia may be elongated (Dionne et al., 2018). On the other hand, extra centrosomes provide additional mature centrioles that act as ectopic basal bodies, resulting in superciliated cells (2–6 cilia per cell), with cilia of normal length (∼4 μm) (Mahjoub and Stearns, 2012).

It appears that transient and sustained CA preferentially lead to multiple cilia and loss of ciliation, respectively. For a better understanding of the relationship between the centrosome and ciliogenesis, briefly, cilia typically assemble in G1/G0 and disassemble before mitosis. Upon cell-cycle exit (e.g., into G0/G1 quiescence), the mother centriole moves to the plasma membrane and interacts with vesicles to form a basal body that nucleates the axoneme. Intraflagellar transport machinery then builds and maintains the cilium by trafficking components along the axoneme. The Golgi apparatus and centrosome maintain close physical proximity, which is essential for trafficking ciliary membrane components, proteins, and centriolar satellites that aid protein delivery.

In ciliogenesis, some kinases (e.g., PLK1) and others control maturation of appendages and the transition from centriole to basal body, and the centrosome organizes microtubules and actin for cilium positioning, polarized secretion, and trafficking, by which it integrates signals and supports the primary cilium as a signaling hub. Extra basal bodies from CA compete for ciliary components, diluting signaling proteins and causing aberrant or inefficient cilia, which disrupt Hedgehog, Wnt, or other pathways, resulting in multiciliated, superciliated, or aciliated cells.

## 3 Genomic instability, inflammation, and aberrant ciliogenesis in cancer and vascular disease

### 3.1 Cancer

#### 3.1.1 Genomic instability

It is known that CA can cause cancer via chromosomal instability. In flies, CA in larval neuroblasts induces multipolar division, leading to aneuploidy and the generation of metastatic tumors upon transplantation into wild-type hosts (Basto et al., 2008). In a mouse model, inducible Plk4 overexpression causes widespread CA, mitotic errors, aneuploidy, and spontaneous tumors in multiple tissues in the presence of intact p53, and tumors exhibit complex karyotypes mirroring those of human cancer (Levine et al., 2017). In humans, CA is a common feature of cancers, occurring at pre-cancerous stages (Pihan et al., 1998) and correlating with tumor malignancy. It is believed that surviving cells with CA propagate unstable genomes that persist across divisions, and the chromosomal instability fuels tumorigenesis, tumor heterogeneity, and progression, which is exacerbated by synergizing with CA-disrupted cell polarity and tissue architecture. The driving role of CA in tumor metastasis is strongly supported by experimental data showing that inhibiting CA via knockdown of signaling molecules substantially attenuated the liver metastasis of colon cancer cells in animal models (Zhao et al., 2026).

#### 3.1.2 Inflammation

In clinical settings, cancer is associated with chronic inflammation driven by endogenous/exogenous physical, chemical, or biological factors, which is blocked/inhibited by anti-inflammatory drugs or non-drug means, showing a causative role of inflammation in tumorigenesis (Greten and Grivennikov, 2019). Mechanistically, inflammatory cells release various factors that induce mutations and thereafter tumor initiation. Proinflammatory cytokines act as direct growth factors, activating NF-κB and STAT3 pathways, which confer resistance to apoptosis, sustain proliferative signaling, and drive stemness of cancerous cells. Inflammation also promotes tumor progression at multiple steps: remodeling of the extracellular matrix by triggering secretion of metalloproteinases, VEGF, and chemokines; inducing epithelial–mesenchymal transition (EMT) and vascularization; creating an immunosuppressive microenvironment by recruiting TAMs/MDSCs that upregulate PD-L1 and inhibit cytotoxic T cells/NK cells; and producing pre-metastatic niches (Greten and Grivennikov, 2019).

It has been suggested that secreted cytokines activate signaling in neighboring cells, inducing invadopodia, actin-rich protrusions, and basement-membrane degradation, which favors EMT and invasion. This non-cell autonomous process occurs even when only a subset of cells has CA, without requiring additional mutations (Arnandis et al., 2018).

#### 3.1.3 Aberrant ciliogenesis

Observational data show that primary cilia are frequently absent in human tumor tissues, suggesting an association between the two. This observation is supported by studies using animal models in which genetic disruption of ciliogenesis (e.g., Tg737/IFT88 deletion) dramatically accelerates tumorigenesis via activation of the mevalonate pathway, which is inhibited by chemical inhibition of the signaling pathway (Gradilone et al., 2013; Seeley et al., 2009). Loss of cilia activates other signaling pathways (e.g., Hh, Wnt/β-catenin, Hippo/YAP-TAZ, Notch, RTK, and TGF-β), alters cell fate, rewrites metabolism toward the Warburg effect (Carotenuto et al., 2023), and creates a pro-tumor microenvironment (Collinson and Tanos, 2025).

All of these changes likely lead to uncontrolled cell proliferation and cancer progression driven by distinct molecular oncogenic drivers (Collinson and Tanos, 2025). Specifically, both aberrant ciliogenesis by CA and cilia loss remove the sensory antenna that integrates growth-repressive signals (Hh, Wnt, Notch, and PDGFR). Cells become insensitive to quiescence cues, thereby driving unchecked proliferation, EMT, and stemness, as observed in the Plk4-induced skin hyperplasia model (Coelho et al., 2015). In contrast, supernumerary cilia dilute signaling molecules (smoothened, Arl13b, receptors), reducing their concentration per cilium, thereby attenuating pathways (e.g., diminished Shh activation). This disrupts polarity and tissue architecture, promoting oncogenic rewiring (Mahjoub, 2013). It appears that the relationship between cilia/aberrant ciliogenesis and cancer development presents a tumor-specific profile. In some cases (e.g., Hh-driven tumors), cilia are required for tumorigenesis, but in most solid tumors, CA-induced cilia loss promotes progression.

### 3.2 Vascular disease

#### 3.2.1 Genomic instability

In diabetic models, DNA damage and a robust DNA damage response occur in endothelial cells and capillary endothelium, with upregulated DNA-PK complex (PRKDC/XRCC5/XRCC6), γH2AX, 8-oxo-dG, and ferroptosis, which leads to increased endothelial permeability, reduced NO bio-availability, and cell dysfunction that can be reversed by pharmacological inhibition of DNA-PK, directly demonstrating causative role of DNA damage (Luo et al., 2025). Causal links between DNA damage-response genes and microvascular complications are also established in genetic studies. Overexpression of CHEK1, which is activated by DNA damage, causally increases diabetic retinopathy risk. Whereas p53-inducible nuclear protein 1 causally elevates the risk of diabetic nephropathy. DNA methylation at specific CpGs further modulates these risks (Liu et al., 2024). Mechanistically, genomic instability and DNA repair activation cause cell-cycle arrest, cell death, or senescence. In ECs, this reduces NO/eNOS, causes microvascular rarefaction, promotes endothelial-to-mesenchymal transition, and accelerates atherosclerosis or basement-membrane thickening (Nagane et al., 2021).

#### 3.2.2 Inflammation

Pathophysiological factors in diabetes initiate and sustain chronic inflammation, which acts as a central driver and amplifier in both micro- and macrovascular complications. In the early stage, inflammation promotes endothelial dysfunction, which is a key event in vasculopathy, leading to tissue damage. In turn, the tissue damage exacerbates inflammation, creating a feed-forward loop that accelerates progression and contributes to “metabolic memory,” in which complications persist post-glycemic control (Li et al., 2023).

The initiating step involves cytokines and NF-κB/NLRP3 activation, which reduce eNOS/NO production, upregulate adhesion molecules, and promote leukocyte adhesion/extravasation. This impairs vasodilation, increases permeability, and initiates microvascular rarefaction. In microvascular complications, cytokine-driven pericyte loss, blood-retinal barrier breakdown, VEGF upregulation, neovascularization, and neurodegeneration underlie retinopathy (Rubsam et al., 2018). Podocyte/glomerular injury, mesangial expansion, tubulointerstitial fibrosis via TGF-β, macrophage infiltration, and chronic injury promote nephropathy (Navarro-Gonzalez et al., 2011). Nerve ischemia and Schwann cell/axonal damage favor the development of neuropathy. Inflammation drives foam cell formation, plaque progression, instability, and rupture. Cytokines promote VSMC proliferation/migration, ECM remodeling, and thrombosis.

#### 3.2.3 Aberrant ciliogenesis

The available evidence suggests that aberrant ciliogenesis contributes to diabetic vascular complications but does not act as a primary or sole cause. It exacerbates endothelial dysfunction and tubular injury by impairing the mechanosensing of shear stress/flow, dysregulating metabolic signaling, and damaging vascular homeostasis. In human DKD kidney biopsies, aberrant ciliogenesis manifests as a dramatic increase in ciliated renal tubular epithelial cells and cilium length, which are positively correlated with DKD histological class and clinical progression, as confirmed in diabetic mouse models (Bai et al., 2022). Endothelial primary cilia sense blood flow/shear stress to regulate Ca^2+^ influx, eNOS/NO production, and vasodilation. Impairment of this mechanosensing promotes hypertension, atherosclerosis, and aneurysms. In diabetic macrovascular contexts, abnormal shear stress impairs endothelial cilia mechanoreceptors and drives procoagulant endothelial states that trigger vascular smooth muscle cell phenotypic transition and remodeling. Specifically, loss of cilia reduces NO bioavailability and causes endothelial barrier failure, PDGF/TGF-β release, VSMC synthetic switch, ECM remodeling, and plaque formation and stiffness (Pala et al., 2018).

## 4 CAID, genomic instability, inflammation, aberrant ciliogenesis, and DAD

As an emerging research area, the existing experimental data on CA and diabetes-associated diseases are limited. Yu et al. (2019) found that an increase in glucose upregulated *O*-GlcNAcylation, which was associated with aberrant ciliogenesis and could explain the phenotypes of cilia loss and shortening in different diabetic animal models. The centrosome was involved because the responsible enzyme was localized to the centrosome and basal body of cilia/centrosome, although CA was not investigated/shown. The authors suggest that aberrant ciliogenesis plays a role in diabetic complications. Ge et al. (2021) reported that diabetes downregulated PDK1 in oocytes, thereby reducing the phosphorylation of PDHE1α at serine 232 and disturbing its enzymatic activity, resulting in abnormal assembly of the meiotic apparatus during oocyte maturation. Because PDK1 regulates centrosome movement (Itoh et al., 2016), the centrosome may play a role in oocyte quality, and CA may adversely affect it. Wang et al. reported that circulating fetuin-A levels were increased in women with GDM, and, *in vitro*, fetuin-A induced CA that blocked primary cilium growth, suggesting an association among CA, aciliation, and diabetes (Wang et al., 2019).

We have shown that the advanced glycation end products (AGEs) induced CA by up-regulating PLK4 and therefore promoted the release of proinflammatory cytokines IL-6, IL-1β, and TNF-α via NF-κB, linking CAID to inflammation. AGEs promoted the metastasis of colon cancer cells via the KLF5–CEP57L1 signaling axis, connecting CAID to cancer metastasis (Zhao et al., 2026). Pathophysiological factors in diabetes also triggered multipolar mitosis, which associates CA in diabetes with chromosomal instability (He et al., 2020). Moreover, AGEs-triggered interaction between CA and cilia loss in renal tubular cells was associated with renal defects resembling those in diabetic nephropathy (Lee et al., manuscript submitted).

## 5 Limitations and future direction

CA can form vicious cycles with DNA damage, inflammation, aberrant ciliogenesis, and others. Mechanisms must exist to quench these cycles and prevent the unlimited escalation of such undesirable events. Otherwise, CA alone would be sufficient to cause a spectrum of rapidly progressing, severe diseases, which does not appear to be the case in reality. Moreover, the weighted contribution of CA to disease pathogenesis should be carefully evaluated to avoid misinterpretation of CA roles. Centrosome aberrations extend far beyond CA; they also include centrosome loss, overelongation, fragmentation, size expansion, and alterations in pericentriolar material (PCM) composition. Whether and how other aberrations contribute to disease remains unknown. There is also evidence that many biomolecules not classically in/associated with the centrosome are localized to the centrosome, and centrosomal proteins can be found outside the centrosome, including those in other organelles, which implicates a complex interaction profile of the centrosome with other subcellular compartments; centrosome function and the adverse effects of centrosome aberration might be far greater than what is understood.

The mechanisms underlying many DADs are poorly understood. Whether DADs have a single pathological pathway or multiple specific pathways remains to be investigated. CA has various adverse effects and is thus a candidate master event in many, if not all, DADs. Notably, DADs encompass more than cancer and vascular complications, and the mechanisms underlying CA actions can extend beyond genomic instability, chronic inflammation, and aberrant ciliogenesis. In addition to diabetes, many physical (e.g., ionizing radiation, mechanical stress/constraint, heat shock, and hypoxia), chemical factors (e.g., cytotoxic compounds, environmental toxins, oxidants, and alcohol), and biological (e.g., aging and infection) factors are also potent triggers of CA. Thus, the centrosome aberrations are likely to play functional roles in a spectrum of pathological conditions and beyond diabetes. The CA-associated diseases may be categorized under a unified concept of “centrosome pathology,” hereby designated as “centrosomopathy”. Importantly, CA occurs through defined signaling pathways, and inhibiting these pathways offers a promising direction for disease management.

## 6 Conclusion

It appears that CA plays a functional role in some diseases associated with diabetes; in cancer and vascular diseases, the underlying mechanisms include, at least, genomic instability, inflammation, and aberrant ciliogenesis. A better understanding of CA/centrosome aberrations and their roles will help develop management protocols for these pathological conditions.

## Data Availability

The original contributions presented in the study are included in the article, further inquiries can be directed to the corresponding author.
